# Differential Hemispheric Lateralization of Emotions and Related Display Behaviors: Emotion-Type Hypothesis

**DOI:** 10.3390/brainsci11081034

**Published:** 2021-08-03

**Authors:** Elliott D. Ross

**Affiliations:** 1 Department of Neurology, University of Oklahoma Health Sciences Center, Oklahoma City, OK 73104, USA; elliott-ross@ouhsc.edu or elliott.ross@cuanschutz.edu; 2 Department of Neurology, University of Colorado School of Medicine, Aurora, CO 80045, USA

**Keywords:** social and primary emotions, hemispheric lateralization, display rules, facial expressions, facial blends

## Abstract

There are two well-known hypotheses regarding hemispheric lateralization of emotions. The Right Hemisphere Hypothesis (RHH) postulates that emotions and associated display behaviors are a dominant and lateralized function of the right hemisphere. The Valence Hypothesis (VH) posits that negative emotions and related display behaviors are modulated by the right hemisphere and positive emotions and related display behaviors are modulated by the left hemisphere. Although both the RHH and VH are supported by extensive research data, they are mutually exclusive, suggesting that there may be a missing factor in play that may provide a more accurate description of how emotions are lateralization in the brain. Evidence will be presented that provides a much broader perspective of emotions by embracing the concept that emotions can be classified into primary and social types and that hemispheric lateralization is better explained by the Emotion-type Hypothesis (ETH). The ETH posits that primary emotions and related display behaviors are modulated by the right hemisphere and social emotions and related display behaviors are modulated by the left hemisphere.

## 1. Introduction

This article will attempt to define, from a behavioral neurology perspective, the role of the right hemisphere in emotions by also addressing the often neglected but equally important role of the left hemisphere in emotions. The article is not intended to be a comprehensive review but rather a focused review by presenting critical research that suggests the left hemisphere modulates *social* emotions and related behaviors whereas the right hemisphere modulates *primary* emotions and related behaviors [1].

## 2. What Is an Emotion and How Is It Measured?

Although there are a number of theoretical models defining what constitutes an emotion, the Perceptual Motor theory (PMT) [2,3] has been most helpful and neurologically relevant in understanding disorders of emotion observed in clinical populations and related animal research. PMT was developed by Leventhal [4,5] because he thought that the four major theories of emotion did not adequately characterize emotions and were contradictory: (1) the Darwinian-evolutionary theory posits that basic or primary emotions are innate because various expressive behaviors associated with emotions are universally recognized and classified across cultures; the basic emotions include happiness, sadness, anger, fear, disgust, surprise (frightful-startle) [6,7,8,9,10,11], (2) the Body Reaction theory most closely associated with James [12] posits that individuals experience an emotion when they became aware of visceral, autonomic and somatic body changes in reaction to an environmental event and that different emotions reflected different patterns of visceral, autonomic and somatic body changes, (3) the Central Neural theory [13,14] defines emotions by identifying various brain regions involved in emotional displays and the generation of internal feeling states and (4) the Cognition-Arousal theory posits that emotions arise when perceptual and cognitive processes cause the individual to become aroused [15,16]. PMT defines emotions as *subjective feeling states* that cannot be assessed directly but are associated with emotional *indicators* that are amenable to measurement. Emotional indicators include: (1) autonomic/hypothalamic responses that may cause changes in heart rate, respiration, lacrimation, sweating, pupillary size, capillary filling and loss of sphincter control, and changes in neuroendocrine secretions, such as cortisol and norepinephrine, (2) somatic motor responses, including arousal, freezing, fight-flight behaviors and species-specific displays involving the face, limbs and torso and non-verbal vocalizations (screams, hoots, etc.) and, in humans, (3) changes in language that include both verbal-linguistic communication (“I am feeling depressed and angry”), a dominant and lateralized function of left hemisphere, and affective-prosodic communication, a dominant and lateralized function of the right hemisphere [6,11,17,18,19,20]. PMT also argues that relying on emotional indicators to assess the emotional state of patients or research subjects may, at times, be very misleading and result in erroneous conclusions. PMT proposes that basic emotional behaviors are universal, consistent with the Darwinian evolutionary theory, as observed in the vocalizations, facial expressions, body and limb movements and autonomic responses of newborns and neonates to environmental stimuli, such as faces, motherese (infant directed speech characterized by excessive affective prosody) [21,22,23], tickling, moving objects, food, sudden noises and other noxious stimuli, and internally generated stimuli, such as colic, hunger and thirst [11,24,25,26]. These innate reflexive behaviors, are the antecedents of primary emotions related to self-preservation. Primary emotions develop initially through a schematic process as the infant begins to cognitively link their reflexive emotional behaviors to specific stimuli and events and learn how to generalize emotional reactions to new situations. A good example of the latter is the behavioral phenomenon of social referencing [27,28]. At around one year of age, infants will look at a parent’s facial expression to help decide how to emotionally react to a novel environmental stimulus, such as a stranger or a new toy. In the conceptual stages of development, emotions become more differentiated and varied as the individual lays down memories of emotional events, often induced by social interactions, appraises the situations and develops coping mechanisms, including anticipation, appropriate levels of arousal and cognitive control of emotional behaviors.

## 3. Are Emotional Indicators Necessary or Sufficient for an Emotional Experience?

In reviewing the literature, one of the more common conceptual disagreements concern whether or not a specific type of indicator is necessary or sufficient for an emotional experience to occur [3,4,17,18]. In the early 1900’s the Body Theory of emotions [12] came under attack [13]. Based on observations that animals who underwent sympathectomy were still capable of producing authentic somatic-motor displays of emotion when appropriately stimulated and observations in sympathectomized patients who reported that they were still able to experience emotions, Canon [13,29] and Bard [30,31] concluded that visceral-autonomic changes were neither sufficient nor necessary for an emotional experience. Furthermore, when human subjects are given pharmacological agents that induce visceral-autonomic reactions, they verbally report either experiencing changes in their physical state without an emotional experience or report experiencing a “cold” “as if” rather than a genuine emotion [32,33]. It has also been reported that when the hypothalamus is stimulated in awake patients undergoing neurosurgical procedures, marked changes in autonomic activity are observed without the patient reporting a concomitant emotional experience [34]. Lastly, if subjects are given pharmacological agents to induce different types of visceral-autonomic reactions and are placed in with group of individuals serving as foils to induce various social-emotional situations, the subjects report experiencing emotions appropriate to the social situation but not specific to the pharmacological agent [15,33].

Another error is assuming that if an emotion indicator is disrupted by a focal brain lesion, the patient is not able to experience emotions. For example, clinical research over the last 40 years has shown unequivocally that acute ischemic lesions involving the right frontal operculum, basal ganglia or medial frontal regions are associated with flattening of affect due to loss of the ability to project emotion into vocal communication, facial expressions and gestures [19,20,34,35,36]. The various affective communication deficits following focal right-brain damage are classified under the term “aprosodia” and the syndromic classification is analogous to the different aphasic syndromes observed after focal left-brain damage [35]. However, patients with affective-communication deficits causing flattening of affect associated with motor-types of aprosodia, are able to experience emotions inwardly (by verbal report), comprehend affective communication of others (by verbal report and formal testing) and are often cognitively aware of that their affective-communication deficits are having a deleterious effect on their interpersonal relationships and psycho-social well-being [34,37]. Even more important, some patients with motor-types of aprosodia may also suffer from a concomitant depression based on their verbal reports of feeling depressed and experiencing anhedonia or suicidal ideation [38,39,40]. Nevertheless, their verbal complaints may be discounted by clinicians because the complaints are not communicated with an appropriate affect.

Another error is assuming that if a patient or research animal displays somatic-emotional behaviors that they are, in fact, experiencing an emotion. In the early 1900’s, research focused on the hypothalamus as a pivotal structure for engendering emotions because it was observed that decorticated or decerebrated animals (cats, dogs) could be stimulated to engage in attack types behaviors as long as their caudal hypothalamus was intact [30]. In addition, electrical or chemical stimulation of certain regions of the hypothalamus were shown to be able to induced somatic motor display behaviors, specifically attack and rage behaviors, and associated autonomic responses in animals with intact forebrains [31,41], also known as the hypothalamic “savage” syndrome [42]. However, it was concluded that these display behaviors and autonomic responses represented “sham” rather than real emotions [11,29,30,31,41,42] because: (1) the display behaviors could be induced in animals that were decerebrated or decorticated, (2) the display behaviors were generalized and not necessarily object directed and could be induced by trivial sensory stimulation, (3) the display behaviors would occur only during electrical stimulation of the hypothalamus in otherwise neurologically intact animals after which the animal would return to its previous activity, as if nothing had occurred, and (4) electrical stimulation of the hypothalamus could not be used as an unconditioned emotional response to induce a learned conditioned emotional response in experimental animals. Lastly, in awake patients undergoing neurosurgical procedures, it has been observed that electrical stimulation of the hypothalamus produces autonomic changes but does not induce somatic motor displays of emotion or verbal reports of an emotional experience [34].

There is also a well-studied clinical condition that supports the idea that somatic-motor displays of emotion are not necessarily indicative that the patient is actually experiencing an emotion. Pathological regulation of affect is a disorder in which the patient will laugh and/or cry in response to trivial environmental stimuli that do not necessarily have any emotional significance [43,44,45]. The emotional displays are very realistic, are not under voluntary control, and have an “all or none” quality. In most instances, based on the patients’ verbal reports, the displays are not associated with an actual emotional experience and do not reflect their actual mood or emotional state. The patients will often complain of their inability to control these unwanted and socially embarrassing behaviors. The most common cause for pathological regulation of affect is pseudobulbar palsy due to bilateral lesions that injure the bulbar neocortical motor regions or their descending connections to the brainstem motor nuclei [46]. To date [18], clinical studies have not resolved if the lesions need only involve the pyramidal (primary) motor cortices or their descending pathways that course through the posterior limb of the internal capsule [47] or whether the lesions also need to also involve the premotor cortices or their descending pathways that course through the genu and anterior limb of the internal capsule [48]. Pathological regulation of affect has also been associated with unilateral or bilateral lesions or epileptic activity involving the basal forebrain, medial temporal lobe, diencephalon, tegmentum and lower brainstem without the presence of pseudobulbar palsy [43,45,49,50,51,52]. Finally, pathological regulation of affect has been reported in several patients after unilateral ischemic lesions involving the right frontal operculum, causing motor aprosodia, who were also suffering from a major depression [39,40]. Although the patients exhibited, at minimum, left upper-motor-neuron facial weakness, they did not have pseudobulbar palsy or structural lesions involving the basal forebrain, medial temporal limbic system, hypothalamus or brainstem. Despite their depression, the patients reported that the unwanted (pathological) emotional displays did not reflect their depressed mood and were not associated with a congruent emotional experience. Interestingly, their pathological regulation of affect rapidly responded to antidepressant treatment well before their depressed mood responded.

The observations reported above regarding pathological regulation of affect suggest that critical lesions or disease processes involving either the neocortical bulbar motor system, basal forebrain, temporal limbic structures or diencephalon release deep seated, innate, reflexive (“sham”) emotional displays [13] that are organized in the brainstem consistent with conclusions reached by Bard [29,30] and Cannon [13,28]. More recent animal-based research has suggested that some innate species-specific emotional displays and associated autonomic reactions are organized in a rostral-caudal pattern within the peri-aqueductal gray matter of the midbrain tegmentum [53,54].

The other clinical condition that supports the concept that the reflexive antecedents of primary emotions reside in the brainstem is hydranencephaly. Hydranencephaly is a syndrome in which the neonate is born without cerebral hemispheres either due to genetic factors, ischemic or hypoxic injury or infection [23,55,56,57]. In the most severe cases (anencephaly), the entire forebrain, including the diencephalon, is missing, as is the calvarium, and the midbrain may be malformed or missing [23,55]. In less severe cases (hydranencephaly), the hypothalamus and other regions of the diencephalon, and portions of the inferomedial occipital and temporal cortices and inferomedial frontal cortex may be preserved with the rest of the forebrain replaced by cerebrospinal fluid surrounded by meninges with an intact calvarium. Neonates with less severe forms of hydranencephaly may survive the post-natal period and live for a number of years with markedly reduced development [57,58]. Some neonates with hydranencephaly may not be diagnosed with the condition until several months after birth when developmental milestones are not met [59]. What is of interest, however, is that neonates with anencephaly, if they survive birth, and neonates with severe hydranencephaly will display many of the innate (reflexive) behaviors associated with primary emotions [13,56].

## 4. What Are the Critical Forebrain Regions for Engendering Emotional Experience?

Once the hypothalamus and brainstem were excluded as pivotal structures for engendering emotions, the forebrain became the focus of research. As suggested by Papez [14], based on anatomical considerations, and later modified by McClean [60,61], based on observations in patients with “psycho-motor” epilepsy who often reported sensory auras that were associated with emotional and other experiential phenomena and visceral-somatic symptoms, the cingulate gyrus, hippocampal formation (limbic lobe), mamillary bodies, anterior thalamic nuclei, amygdala and orbitofrontal cortices were thought to be the key structures for emotional experience. The critical research for determining which structure was essential for an emotional experience was initiated by Penfield and associates [62,63] who pioneered the technique of electrically stimulating the forebrain in awake patients undergoing neurosurgical procedures. They found that electrical stimulation of the temporal limbic regions and anterior temporal and inferior frontal cortices induced patients to report emotional and other experiential phenomena, such as hallucinations, delusions, paranoia, alterations in the sense of time (déjà vu and jamais vu), heightened drive states and vivid memories of past events. What binds all of these events together is that the patients report that they were very real experiences despite being induced by electrical stimulation. More contemporary research [64,65,66], using arrays of cortical and depth electrodes, established that reports of experiential phenomena occur only if electrical stimulation of the inferior frontal and anterior-lateral temporal cortices is propagated into the amygdala/hippocampus or if the amygdala/hippocampus is stimulated directly. Behavior research in both humans and experimental animals has established that bilateral lesions involving the hippocampal formation [67,68], specifically the CA1 region of the hippocampus proper and the perirhinal and hippocampal cortices [69,70,71,72,73], produces severe and permanent loss of the ability to learn new information (anterograde amnesia) and retrieve information from the recent past (retrograde amnesia) that is multimodal [74]. Bilateral lesions of the amygdala, however, do not cause memory loss or enhance memory loss following lesions of the hippocampal formation in monkeys [75] but are associated with profound hypoemotionality and loss of autonomic reactivity to external stimuli [76,77,78,79,80,81]. In contrast, bilateral lesions of the hippocampal formation are not associated with hypoemotionality or loss of autonomic reactivity to external stimuli [79,81].

## 5. What Is the Amygdalae’s Role in Emotion and Memory?

**Figure 1:**
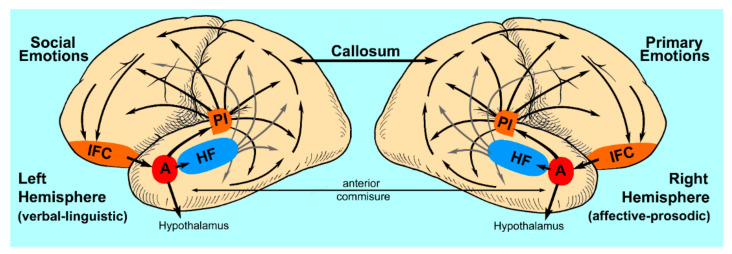
A highly schematized drawing depicting the emotional and mnestic pathways and neural networks that are involved with emotions, memory, executive functions and cognitive appraisal that ultimately furnish feedback to the amygdala and hippocampal formation (A = amygdala; HF = hippocampal formation; IFC = infero-frontal cortex; PI = posterior insula). Not shown are the ipsilateral parallel convergent inputs from the primary visual, somatosensory and auditory cortices that convey exteroceptive information to the amygdala and hippocampal formation for generating an emotional experience and laying down memories that store both the factual and emotional aspect of the exteroceptive event [1,17,18,20,74]. The nodal point for processing exteroceptive information into memory is the hippocampal formation, however the mnestic information is actually stored in neocortex in a distributed neural network (multiple gray arrows). The nodal point for generating an emotional reaction to exteroceptive information is the amygdala but to experience and remember the emotional event requires the participation of the posterior insula and its associated neural network (multiple black arrows). The amygdala also has the capability of enhancing or diminishing the strength of factual memories stored in neocortex via its efferent connections to the hippocampal formation (short black arrows). The factual and emotional memories stored mainly in the posterior neocortices can, in turn, be relayed to the prefrontal neocortices for complex cognitive processing involving executive control of behavior (lateral pre-frontal areas) and cognitive appraisal of emotions (inferior and medial pre-frontal regions) that, in turn, can furnish feedback to the amygdala and hippocampal formation via efferent connections from the inferior frontal cortex (IFC). The emotional and mnestic information that is processed by each hemisphere can be shared via distributed connections that travel through the corpus callosum and anterior commissure.

Although the amygdalae appear to be the critical forebrain region for engendering emotions, the next question to ask is: are the amygdalae sufficient to generate an emotional experience? From an anatomical perspective, the amygdalae sit at a unique cross road in the primate brain (Figure 1) since each has ipsilateral reciprocal connections with the hypothalamus, various brainstem nuclei, including the periaqueductal gray, substantial nigra, locus coeruleus and dorsal raphe, the hippocampal formation and insula. The amygdalae also receive ipsilateral afferent input from the anterior temporal, inferior frontal and medial frontal cortices and have ipsilateral efferent projections to the medial and dorsolateral frontal, inferior-medial occipital and temporal neocortices [82,83]. In addition, there are four other neuroanatomical issues that need to be emphasized to fully understand the amygdalae’s role as the nodal point of a distributed cortical neural network underlying the experiential aspects of emotions [1,17,18,74].

The amygdala-hippocampal formation receives exteroceptive sensory information regarding the environment via parallel-convergent inputs from the primary visual, somatosensory and auditory neocortices [1,74,84,85,86,87,88]. The input pathways are multisynaptic in primates [84] but, at least in humans, there is evidence that a more direct pathway exists as part of the inferior longitudinal fasciculus for vision [89]. Within the medial temporal limbic system, the amygdala has strong (efferent) projections to the ipsilateral hippocampal formation but receives only weak (afferent) projections from the hippocampal formation [88]. Thus, the amygdala is in a unique position to modulate (amplify or diminish) the strength of mnestic processing of exteroceptive sensory information by the hippocampal formation that is ultimately stored as a distributed neocortical network based on sensory attributes [1,17,74,90]. The densest neocortical projections from each amygdala are directed to the ipsilateral posterior insula, which, in turn, has wide spread connections to the rest of the cortical mantel [88,91,92]. Thus, each amygdala is able to suffuse ipsilateral hemispheric cognitive processes with emotional and affective tone [1,74]. Finally, in primates and especially in humans, each temporal limbic system is overwhelmingly connected to the sensory and cognitive processes modulated by the ipsilateral hemisphere and has only very sparse direct interhemispheric connections or functional affiliations with the contralateral temporal limbic system [1,93,94,95,96,97,98,99,100]. Therefore, any interactions between the right and left amygdala-hippocampal formation must occur indirectly, via multisynaptic connections, that involve the ipsilateral neocortex, corpus callosum or anterior commissure and contralateral neocortex [1,17,18]. In humans, cognitive functions, especially those related to language [20,22], are lateralized early in life [101], implying that each hemisphere’s underlying mnestic processing of exteroceptive information by the hippocampal formation that drives the development of cognitive functions is also differentially lateralized. Thus, it would be reasonable to assume that emotions and related experiential phenomena generated by each amygdala, including their cognitive elaboration by ipsilateral neocortex and associated autonomic and somatic motor indicators, should also be differentially lateralized [1].

The publications by Kluver and Bucy [102,103], describing psychic blindness, hyperorality and hypoemotionality in monkeys after bilateral anterior temporal removals, initiated extensive research into the underlying neuroanatomy of both memory and emotions [74]. Subsequent research in monkeys established that bilateral lesions of the anterior-inferior temporal neocortex (area TE) caused severe deficits in the acquisition of new visual discriminations [104,105] without the other behavioral components of the Kluver-Bucy syndrome or deficits in the acquisition of new tactile [106] or auditory discriminations [107]. Further research demonstrated that bilateral lesions involving either the anterior-superior temporal [108] or lateral regions of the posterior-superior temporal gyrus that spared the planum would cause severe loss of the ability to learn new auditory discriminations that did not affect the ability to learn new visual discriminations [109]. Finally, it was demonstrated that bilateral lesions of the posterior parietal cortex would induce loss of the ability to learn new tactile but not visual discriminations [106,110]. The sensory-specific disorders of learning described above were shown to be an amnestic rather than an agnostic type of memory loss since the lesioned animals were able to retain over-learned sensory discriminations but not recently learned (pre-operative) discriminations [74,110,111,112,113,114]. Based on his disconnection approach to brain-behavioral relationships, Geschwind [115] suggested that the sensory-specific visual learning deficits and hypoemotionality in monkeys after bilateral TE ablations were due to a visual-temporal limbic disconnection syndrome rather that the TE ablations themselves, a hypothesis that was confirmed by Horel and Misantone [116]. They were able to produce sensory-specific visual learning deficits (and sensory-specific visual hypoemotionality) in monkeys after placing coronal white matter cuts that transected the white matter tracts that course longitudinally through the posterior basolateral temporal lobe that are part of the ventral stream and include the inferior longitudinal fasciculi proper and multisynaptic U-fiber pathways [84,89,117].

In 1980, Ross [87] published the first clinical description of sensory-specific visual amnesia in two patients who had suffered bilateral posterior cerebral infarctions involving, at minimum, the inferior longitudinal fasciculi, and varying amounts of the medial-inferior occipital and inferior-posterior temporal cortices. Neither patient, by CT scan, had an ischemic injury involving the anterior temporal lobes or the medial temporal limbic regions and neither patient demonstrated visual object agnosia on formal testing. Both patients had preserved ability to acquire new right and left tactile memories and verbal and non-verbal auditory memories. In a subsequent publication, Bauer [118] presented a third patient with sensory-specific visual amnesia that was the result of bilateral inferior occipito-temporal hematomas secondary to trauma. Initially, the patient had a classic multi-modal amnesia affecting visual, tactile and auditory modalities [74,87,94,119] that partially recovered over 6 months, leaving him with a sensory-specific visual amnesia. However, his most striking behavioral symptom was that “… he could no longer become emotionally or sexually aroused by visual stimuli, and that his visual world had become drab and uninteresting. In contrast, he could experience full emotional responses to stimuli presented in other modalities” (p. 702). On formal testing, the patient was found to have loss of autonomic reactivity to emotionally provocative visual, but not auditory or tactile, stimuli. The patient’s signs and symptoms, including loss of autonomic reactivity, were attributed to a visual-limbic disconnection syndrome that deprived the amygdalae and hippocampal formations from receiving and processing visual, but not somatosensory or auditory information. Two similar case of sensory-specific visual hypoemotionality have been reported after bilateral inferior temporo-occipital lesions [120,121,122], however, memory functions were assessed using standard auditory-verbal stimuli rather than modality specific stimuli designed to detect sensory-specific or fractional amnestic disorders [74,87,94,119]. Interestingly, there has been one report of visual hypoemotionality following a strictly right sided temporo-occipito-parietal intraparenchymal hemorrhage as a complication of interstitial laser ablation of a right temporal tumor that, based on diffusion tensor MRI imaging, resulted in interruption of the right inferior longitudinal fasciculus [123].

In addition to describing sensory-specific visual amnesia, Ross [94] also described patients with fractional amnestic disorders as a result of unilateral posterior cerebral artery infarctions that involved the medial portions of the occipital lobe causing a contralateral hemianopsia and the posterior medial portions of the temporal lobe that included the hippocampal formation (Cases 2,3). The patient with a left-sided lesion demonstrated a right, but not a left, loss of tactile recent memory and a verbal, but not a non-verbal, loss of auditory recent memory. The patient with the right-sided lesion demonstrated a left, but not a right, loss of tactile recent memory and a verbal, but not a non-verbal, loss of auditory recent memory. As expected, neither patient had difficulties with visual recent memory in their intact ipsilateral visual field. Unfortunately, the patients were not assessed for possible “fractional” hypoemotionality as a companion symptom since these cases were published prior to the description of sensory-specific visual hypoemotionality as a companion symptom to sensory-specific visual amnesia [118]. The possibility of fractional hypoemotionality would only occur if the posterior cerebral artery infarction also involved the amygdala, in addition to the hippocampal formation. This is unlikely in humans because the anterior choroidal artery, a terminal branch of the internal carotid artery, serves as the main vascular supply to the amygdala and also the head and very anterior portions of the hippocampal formation [124,125,126]. Thus, if fractional hypoemotionality were to be described clinically, it would most likely be the result of a unilateral anterior choroidal infarction either in conjunction with a fractional loss of recent memory, if enough of the hippocampal formation is injured, or as a stand-alone phenomenon, if the infarction did not involve the hippocampal formation. Although isolated anterior choroidal artery infarctions are relatively rare [126], they offer a potential means for clinicians to directly assess for emotional lateralization by identifying patients with acute unilateral infarctions that involve the amygdala [94].

The next issue concerns whether or not the amygdalae are sufficient for engendering an emotional experience and associated memories or do they serve as crucial nodal points for a distributed cortical network that underlies emotional experience and associated memories (Figure 1), similar to the role of Wernicke’s area for comprehension of the verbal aspects of language or the hippocampal formation for mnestic functions [1,18,20,74]. The clinical syndrome that suggests the amygdalae are part of a distributed cortical network is pain asymbolia, first described in the early 1900’s [18,74]. Pain asymbolia [127,128,129,130] is an acquired condition in which the patient has trimodal loss of emotional reactivity to threatening somatosensory, auditory or visual stimuli. When presented with threatening stimuli, such as wielding swords, cutting their skin with knives, loud noises or verbal threats of violence, which (I assume) were most likely delivered with appropriate affective-prosodic intensity to ensure that the verbal threats were realistic, the patients do not engage in appropriate defensive or avoidance behaviors, either initially or after repeated threats, indicating that there is no learning effects and they are able to dispassionately describe the threatening or noxious aspects of the stimuli, suggesting the condition is not an apperceptive or associative type of agnosia. If a patient is cut on the arm, on repeated attempts to cut their arm, the patient may actually proffer his limb towards the examiner rather than withdraw it. Pain asymbolia is more common after right versus left brain lesions and was initially thought to be associated with lesions that localize to the inferior parietal region. However, a more recent study by Berthier and colleagues [130] found, based on CT scans, that injury to the posterior insula, a neocortical region that receives the densest efferent output from the amygdala and has widespread afferent connects with the neocortex [88,91,92], was critical for inducing the syndrome of pain asymbolia. In addition, they reported that the patients had appropriate autonomic reactions to threatening stimuli even though they did not show appropriate emotional or avoidance responses to the stimuli. This strongly suggests that the threatening stimuli were processed by the amygdalae and that pain asymbolia represents a sensory-limbic post-processing deficit [18,74]. Thus, it appears the amygdalae are not sufficient to engender emotional experiences and associated memories but do so as part of a distributed cortical neural network (Figure 1).

## 6. Differential Hemispheric Lateralization of Emotions and Associated Behaviors

Currently, there are two major hypotheses regarding emotional lateralization, the Right Hemisphere Hypothesis (RHH) and the Valence Hypothesis (VH) [131,132]. The RHH posits that emotions and associated display behaviors are a dominant and lateralized function of the right hemisphere. The hypothesis is based on the observations that right, as opposed to left, brain damage impairs: (1) the perception and comprehension of emotional stimuli across multiple channels, including affective prosody and facial expressions, (2) the expression of emotions through multiple channels, including affective prosody and facial expressions, resulting in a flattened affect, and (3) autonomic-arousal responses to emotional stimuli [1,19,20,34,35,36,37,133,134,135,136,137,138,139,140,141]. However, as outlined in Section 2. above, the RHH is based on loss of emotional indicators rather than loss of the ability to experience emotions, which, if explored clinically, is often preserved in patients with focal right brain damage [34,37,38,39]. The other problem with the RHH is that each hemisphere has an amygdala, hippocampal formation and posterior insula, implying that the left hemisphere should have the neurological ability to modulate emotions and related behaviors, including autonomic responses, that is complimentary to the right hemisphere, analogous to the right hemisphere modulating the affective-prosodic aspects of language and communication and the left hemisphere modulating the verbal-linguistic aspects of language and communication [20]. In many respects, the VH attempts to address this issue. The VH posits that negative emotions and related behaviors are modulated by the right hemisphere and positive emotions and related behaviors are modulated by the left hemisphere [131,132]. It was derived based on initial observations in patients undergoing left and right-sided Wada tests in order to determined cerebral lateralization of language and memory functions before they underwent neurosurgical resections to treat intractable epilepsy [142,143,144,145].

The Wada test is accomplished by hand injecting sodium amobarbital into the right carotid artery and, after recovery, into the left internal carotid artery. The amobarbital temporarily anesthetizes the ipsilateral hemisphere which allows clinicians to briefly test language and memory functions of the non-anesthetized hemisphere. In patients with standard arterial circulation in which each anterior cerebral artery is a branch of the ipsilateral internal carotid artery and each posterior cerebral artery is a branch of the basilar artery, the effects of amobarbital are manifested almost exclusively in the anterior circulation of the hemisphere (anterior choroidal, middle cerebral and anterior cerebral arteries) and most prominently in the areas most proximal to the internal carotid artery [1,146,147,148]. However, this distribution may be altered if the patient has an anomalous cerebral circulation, such as the azygous variant (prevalence of 2–4%) where both anterior cerebral arteries branch off one internal carotid artery and the fetal origin variant in which one (prevalence of 10%) or both (prevalence of 8%) posterior cerebral arteries branch off the ipsilateral internal carotid artery [149]. In patients with standard cerebral circulations, the posterior Sylvian regions and the borderzone areas of the parietal, occipital and temporal lobes may escape barbitization and explain why verbal comprehension is often preserved during left-sided Wada tests [143,147] with similar results for affective-prosodic comprehension after right-sided Wada tests [1,148]. The areas of the hemisphere irrigated by the posterior circulation (posterior cerebral artery, thalamoperforating and thalamogeniculate arteries) are unaffected and include the mesial and inferior occipitotemporal regions and thalamus [125,150].

Initial observations reported that left-sided Wada tests often induced a depressive-catastrophic (negative) behavioral reaction (crying, pessimism, guilt, despair) whereas right-sided Wada tests often induced a euphoric-maniacal (positive) behavioral reaction (laughing, smiling, optimism, inflated sense of well-being), at a point in time when the hemiplegic and other motor effects of the amobarbital have abated [143,144,145,149,151]. This led to a number of EEG-based studies that demonstrated increased EEG activation of the left hemisphere if subjects were exposed to stimuli portraying positive emotions and increased EEG activation of the right hemisphere if subjects were exposed to stimuli portraying negative emotions [131]. Most recently, the VH now includes the concept that the right hemisphere is involved with emotions that induce withdrawal behaviors and the left hemisphere is involved with emotions that induce approach behaviors and that there may also be a motivational factor [131,132,145,152,153,154].

### 6.1. Social Emotions

Although both the RHH and VH are supported by extensive research data, they are, in fact, mutually exclusive, suggesting that there may be a missing factor in play that may provide a more accurate description of how emotions and related behaviors are lateralization in the brain [1,17,18]. Emerging data, that are not yet well appreciated clinically, has provide a much broader and inclusive perspective of emotions by embracing the concept that emotions and related behaviors can be dichotomously classified into primary and social types [1]. As outlined in Section 2, primary (basic) emotions and related displays are developmental derivatives of innate reflexive neonatal reactions to internal and external stimuli that are universally recognized across cultures-happiness, sadness, anger, fear, disgust, surprise (frightful-startle) [6,7,8,9,10,11]. Primary emotions are related to self-preservation and, except for happiness (elation), are negative in valence and associated with withdrawal, flight or fight types of responses [10,11]. In contrast, social emotions [10,11,155,156,157,158,159] are acquired through parental socialization of toddlers and young children to behave in a manner that is socially acceptable and when toddlers and young children engage in various social interactions during play, school and religious-cultural activities. Although the human brain is innately wired to learn and cognitively modulate social emotions, similar to the acquisition of the verbal-linguistic aspects of language, what is acquired is culturally dependent and, consequently, not associated with universally recognized display behaviors. Examples of social emotions include admiration, anger, contempt, delight, embarrassment, envy, empathy, gratitude, guilt, jealously, love, pity, pride, scorn and shame. In infancy, social emotions are thought to be a derivative of the biological drive of attachment but, in later in development, the motivation is to gain the approval, affection and admiration of others [1,10,11,160,161,162]. Although social emotions may be positive or negative in valence, they are associated with culturally-dependent “display rules” [155,163,164,165,166,167,168,169], whereby children learn to cognitively manipulate their facial expressions (and voice) to make their emotional displays socially acceptable through (1) intensification: enhancing a felt display, (2) minimization: dampening a felt display, (3) masking: displaying no emotion when one is felt, (4) simulation: displaying an emotion when none is felt, (5) dissimulation: displaying a different emotion than the one that is felt, and (6) qualification: displaying a different emotion on the upper versus lower face [169,170]. This, in turn, allows for amicable (positive or approach) types of social interactions to occur between individuals even if the emotional displays are at odds with their internal emotional animus. Thus, in acquiring display rules, children also gain the means to engage in deceitful or false behaviors [164,169,170,171,172,173].

In 1980, Buck and Duffy [174] published a seminal paper that provided strong evidence to suggest that display rules, and consequently social emotions [1], were a lateralized function of the left hemisphere. They analyzed the facial expressions and upper body gestures in five research groups: patients with Parkinson disease, patients with right hemisphere strokes that resulted in at least a contralateral hemiparesis with or without sensory loss, patients with left hemisphere strokes that resulted in a right hemiparesis and aphasia, age-equivalent normal adults and preschool children. The research groups were shown slides that varied from being very positive, so as to elicit a social emotional response, to being very negative, so as to elicit a primary emotional response [1] and included pictures of familiar people (very positive), pleasant scenery (positive), unusual photographs that were somewhat disconcerting because of photographic effects, such as double exposures (negative), and very unpleasant pictures, such as a starving child or a crying woman (very negative). The subjects’ emotional responses to each slide were covertly videotaped and judges were asked, based on the subjects’ expressive behaviors, to identify which type of slide was being viewed. Densely aphasic could be assessed because the research paradigm did not require verbal instructions or verbal responses. The age-equivalent adults and preschool children displayed a distinct sculpted pattern of emotional responses with the most pronounced and identifiable responses associated with the familiar person slides (~55%) that incrementally decreased for the scenic slides (~46%), unusual slides (~40%) and unpleasant slides (~24% or at chance levels). In contrast, patients with left-side strokes did not sculpt their responses: familiar person slides (~44%), scenic slides (~50%), unusual slides (~47%) and unpleasant slides (~44%). In contrast, patients with right-side strokes had overall blunting of their responses but still maintained a sculpted response pattern: familiar person slides (~45%), scenic slides (~35%), unusual slides (~30%) and unpleasant slides (~25% or at chance levels). Parkinson patients preformed similar to the patients with right-sided strokes patients but were overall less expressive. The sculpting of emotional responses in adults and children was attributed operationally to display rules, which were lost in patients with left-sided strokes but retained in patients with right-sided strokes and Parkinson disease.

### 6.2. Cerebral Lateralization of Emotions: Emotion-Type Hypothesis (ETH)—A Serendipitous Discovery

This section is written in the first person in order to accurately convey to the reader how the ETH came into existence since it was formulated based on a serendipitous, inductive, research discovery and not through the traditional, hypothesis-driven, deductive methods of research [119]. In 1988, my colleagues (Richard Homan, M.D., Neurologist-Epileptologist; Jerold Edmondson, Ph.D., Linguist; G. Bert Seibert, Ph.D., Statistician) and I [148] published a research study to determine the acoustical underpinnings of affective prosody by testing and tape recording the spontaneous and repetitive responses of five strongly right-handed patients before, during and after undergoing a right-sided Wada test. Because of time limitations, prior to the right-sided Wada test, I assessed their affective prosody using a brief repetitive task in which they were asked to imitate six emotions. I also probed the patients to recount an affectively noteworthy emotional life experience in order to obtain a sample of spontaneous affective prosody. The patients were assessed during and after the right-sided Wada test using the exact same repetitive task and they were also asked to recall the emotional life experience identified in the pre-Wada evaluation. The patients could not undergo testing during their left-sided Wada test because all had severe non-fluency and most had aphasic comprehension deficits. After collecting five patients for the acoustic paper [148], Richard Homan and I continued testing six more patients that eventually comprised the research group for the ETH publication [1]. The following excerpts are chosen to highlight our findings regarding changes in emotional recall during the right-sided Wada test.

The very first patient tested prior to the right-sided Wada test [1] recounted his last car accident in which he ran off the road and stuck a tree. He was thrown from the car and sustained a momentary lost consciousness but was not seriously injured. His initial memory of the event was seeing his car “squashed like an accordion.” When asked to recall his emotional state, he instantly and emphatically replied “I was scared, scared to death, I could have run off the road and killed myself or someone else I was really scared.” During the right-sided Wada test, he recalled the factual aspects of the accident correctly. However, when asked about his emotional reactions he replied “Silly … silly.” When questioned if he were afraid or frightened, he replied “Oh … maybe a little bit.” As the amobarbital began to wear off, he was again asked about his emotions after the accident and replied “I felt kind of stupid.” When asked directly if he felt frightened or scared his response was “it was a bad accident but … it was not anything that was physically damaging to anyone.” When asked directly he never admitted to being either scared or frightened. After the Wada test, he was asked to once again recall his last car accident which reverted to the pre-Wada recall. He emphatically pointing out how “scared” he was and denied that he had felt either “silly” or “stupid”. The dramatic change in the patient’s emotional recall regarding his car accident during the right-sided Wada test was quite unexpected and certainly not planned for because the research goal was to acoustically analyze affective prosody not assess emotional memory. Also, the change in memory was perplexing as it did not fit with either the RHH or VH of emotional lateralization. If the RHH was correct, the right-sided WADA test should have erased or minimized all emotional recall of the event. If the VH was correct, the emotional memory that was repressed but stored in the patient’s intact left hemisphere that was brought into the conscious foreground by the right-sided Wada test should have been positive rather than negative.

The second patient [1] recalled a car accident due to a seizure. He hit a curb that caused him to veer into a yard, strike a tree, swerved back into the street, eventually coming to rest on a front lawn in which several children were at play. When asked about his emotional response, almost before the question was finished, he replied very emphatically “I was scared, I was upset pretty bad about tearing the car … I was very scared mostly when I saw the kids that were playing. that scared me more than anything else because I realized I could have killed them.” During the right-sided Wada when asked to recall his car accident his first response was “I could have killed about five little children.” He then filled in some detail about the accident. When asked to describe his emotions after the accident, he replied “I was depressed … about tearing up my car, new car.” When asked about any other feelings he experienced, after some delay he finally said “I was afraid that I could have killed some little children.” Despite prompting, he never admitted to being either scared or very scared. After the Wada test his emotional recall reverted to the pre-Wada condition. Again, the changes in memory recall during the right-sided Wada test did not fit with either the RHH or VH. Prior to the Wada test he recalled two contrasting negative emotions: being very scared that he could have struck some children with his car and being upset about tearing up his new car. During the Wada test, he still recalled that he almost hitting some children but instead of reporting being very scared, his emotional memory was minimized and decathected to being afraid, most likely reflecting a repressed left hemisphere perception of the event. In contrast, the emotional memory of being very upset about wrecking his car was enhanced to being depressed, suggesting that both negative emotional memories were the left hemisphere’s perception of the event.

The fourth patient [1], near the end of his pre-Wada evaluation, recalled that several physicians had missed diagnosed his peculiar auras and they did not truly appreciate his problem until he had an overt seizure. When asked about his emotional reactions to his illness, he replied in both words and tone of voice that he was very “angry” and “frustrated.” During the right-sided Wada test when asked to recall his emotional frustration his response was “sorry, I felt sorry for people that they had so much trouble finding out what was wrong.” He denied being either angry or frustrated despite prompting. Post-Wada his emotional recall reverted to the pre-Wada condition. Again, the changes in memory recall during the right-sided Wada test did not fit with either the RHH or VH. Although the denial of being angry and frustrated regarding his seizure evaluation during the Wada test would be consistent with the RHH, his repressed recall of feeling sorry for the people trying to diagnose him is not. Feeling sorry, a negative type of emotion, was not consistent with the VH but could represent an empathetic social-type of emotion.

The fifth patient [1] during the pre-Wada interview recounted that when she was growing her siblings and school mates thought she was retarded and would tease her by calling her “stupid” and “dumb.” When asked how she felt about the teasing, her response was very emphatic, “mad and angry.” During the right-sided Wada test she recalled that children, including her siblings, always “made a lot of fun” about her seizures. When further question, she eventually admitted that the teasing made her feel “embarrassed” and denied that it caused her to feel angry or mad. After recovery from the Wada test, her emotional recall reverted to the pre-Wada state and she vigorously denied that she ever felt embarrassed. Again, the change in emotional recall during the right-sided Wada test did not fit with either the RHH or VH. Feeling embarrassed is a negative emotion but could be viewed as a social-type of emotion.

The seventh patient [1] recalled a verbal fight with his girlfriend that became physical. He then related that he became very “scared” because he felt that the situation had become dangerous. He finally “told her, if you don’t stop this I am going to kill you” and the fight came to an ended. During the right-sided Wada test he remembered the details of the fight, including what he said to end it, but did not admit that he was scared, even on direct questioning. After the Wada test his emotional recall of the fight reverted to becoming scared. Although the denial of being scared during the Wada test would be consistent with the RHH his retained recall that he stopped the fight by verbally threatening to kill his girlfriend would not be consistent with either the RHH or VH.

After reviewing the data, we thought that we had uncovered a novel method for studying repressed memories. However, I knew intuitively that we had accidentally stumbled on something very important regarding emotional lateralization and, therefore, delayed the publication of our results until I had defined exactly what it was that we had discovered. In April of 1991, I was invited to give a lecture on the neurology of affective communication at colloquium held at the University of Texas in Austin. One of the invited speakers was Ross Buck, Ph.D., a Social Psychologist whose research involved emotions and motivation [10,168,174]. We instantly realized that we had common research interests and shortly thereafter I invited him to visit Fargo, ND, to deliver a lecture to the Department of Neuroscience (University of North Dakota School of Medicine) and discuss potential collaborative research opportunities. During his visit, I showed him the transcriptions of the tape recordings that were made before, during and after the right-sided Wada test. He poured over the transcriptions and took copious notes. After about a half an hour he looked up and stated that nearly all of the pre-Wada emotional memories could be categorized as primary emotions and that during the right-sided Wada test most patients minimized their recall of the primary emotional memory with three patients actually outright denying their pre-Wada primary emotional memory of the event; four patients switched their recall from a primary to a social emotion; one patient enhanced his social-emotional recall and diminished the primary emotional recall of the life event; one patient appeared to switch his recall from one primary emotion to a different (negative) primary emotion and one patient did not alter his emotional recall during the right-sided Wada test. (I was not able to elicit an emotional memory in one patient either before or during the Wada which was attributed to possible alexithymia [1]). My first reaction was “what is a social emotion”, a concept that I had never encountered in the behavioral neurology, neuropsychology or neuropsychiatry literature. He explained what social emotions were and how they development during childhood, including display rules. As soon as I read the relevant literature, we (Ross, Homan, Buck) decided to publish the research under the title “Differential hemispheric lateralization of primary and social emotions: Implications for developing a comprehensive neurology for emotion, repression, and the subconscious” [1].

A few comments regarding the research methodology and results are needed. The deductive research goal was to acoustically assess production of affective prosody before, during and after a right-sided Wada test [148], not emotional recall. The finding that almost all of the pre-Wada emotional memories were primary and not social is due to the fact that when I was interviewing the patients prior to the Wada test, I encouraged them to remember a life event that caused them, for example, to become angry or fearful (standard “primary” emotions) in order to obtain a sample of spontaneous speech that was affectively driven. At the time, I was not aware of social emotions as a separate entity and, therefore, did not ask patients to recall a social-emotional life event that caused, for example, embarrassment, jealousy or surprise. Nevertheless, two patients, in addition to vividly recalling their “primary” emotional reaction, also mentioned a second emotional feature of the life event that, based on the change in recall during the right-sided Wada test, indicated that the second emotional feature was left hemisphere based. For example, patient 2 (see above) reported that he was very scared about possibly hitting some children when he lost control of his car and, as an aside, commented that he was also upset about tearing up his car. During the right-sided Wada test his spontaneous recall of the event was being depressed rather than being just upset about tearing up his car and only when questioned directly did he admit to being afraid rather than being just scared or very scared that he could have killed some children. However, the responses that solidified our impression that social emotions were lateralized to the left hemisphere involved the three patients who changed their spontaneous memory of the event from a primary to a social emotional recall and verbally denied their primary emotional recall of the event. Except for the one patient who may have been alexithymic, all patients either minimized or outright denied their primary emotional recall when questioned during the right-sided Wada test, an observation that strongly supported our impression that primary emotions are lateralized to the right hemisphere. The minimization of the primary emotional recall on verbal questioning during the right-side Wada test most likely elicited from the left hemisphere either a social-emotional memory or a decathected verbal-cognitive explanation of the event. The last issue concerns how language and the role of the callosum in integrating the verbal-linguistic and affective-prosodic aspects of communication shaped and influenced our interpretations when developing the ETH (Figure 1). Prior to the Wada test, the recall of the life event elicited a verbal description of the event (left hemisphere) that included both the factual statements and the right hemisphere’s emotional reaction to the event that was further supplemented by an appropriate affective-prosodic response (right hemisphere) [1,18,19,20,175,176]. During the right-sided Wada test, the verbal recall continued to elicit the same factual descriptions of the life event but what changed was the emotional description, suggesting that the left hemisphere no longer had access, via callosal connections, to the primary emotional memories stored in the right hemisphere but still had access to the emotional memories stored in the left hemisphere that were either social in quality or a decathected version of the pre-Wada primary emotional recall. The verbal recall, even when communicating emotional information, was done in a flat monotone voice that was devoid of affect, suggesting that the right hemisphere was no longer able to access, via callosal connections, the emotional memories stored in the left hemisphere and insert the appropriate affective prosody to supplement the patient’s verbal communication.

### 6.3. Are There Other Lines of Research That Support the Emotion-Type Hypothesis?

To date, there has been no research that directly investigates the basic premise of the ETH that social emotions and related displays are lateralized to the left hemisphere and primary emotions and related displays are lateralized to the right hemisphere. However, there has been considerable research regarding the role of the prefrontal cortex (PFC) in complex, higher-order, cognition that includes social-emotional and executive types of functions [177,178]. The PFC, from a neuroanatomical perspective, matures later in human development compared to the posterior neocortical regions of the brain with the lateral PFC being the last to mature [177,179]. Each PFC receives extensive ipsilateral afferent inputs from the parietal, occipital and temporal regions [177,178], which allows it to cognitively integrate and synthesize exteroceptive mnestic and related emotional information that was initially processed through the temporal limbic system and posterior insula before being stored in neocortex (Figure 1) [1,18,74]. The right and left PFC also have extensive ipsilateral efferent connections to the temporal limbic system (especially the amygdala [180]), basal ganglia, thalamus and various neocortical regions including the premotor and primary motor areas [177,178]. The inferior and medial regions of the PFC appear to be mainly involved with processing, interpreting, and appraising social-emotional information that also includes comprehension of affective prosody and identification of facial expressions [19,181,182] and the regulation of emotional behaviors. In contrast, the dorsolateral regions appear to be mainly involved with executive-types of cognitive functions to guide appropriate motor responses, including the propositional (verbal-linguistic) aspects of language [177,183]. The dorsolateral and inferomedial regions also have reciprocal connections so that higher-order social-emotional cognition is able to influence executive functions and vice versa [177,184,185].

There has been only one research study designed to indirectly address the validity of the ETH. Shamay-Tsoory and colleagues [186] assessed whether recognition of basic (primary) emotions was preferentially processed by the right PFC versus whether recognition of complex (social) emotions was preferentially processed by the left PFC. They tested 17 patients who had sustained injury to the left PFC and 10 patients with injury to the right PFC, comparing their performance to 47 healthy age-matched controls. The stimulus set was derived from photographs of facial expressions that were classified as either basic or complex emotions. The photographs were cropped to just show the eye region. Both the basic and complex emotional stimuli were further stratified based on whether they were positive or negative in valence. They also ran a second experiment in which the stimuli were presented tachistoscopically to either the right or left visual fields of 55 healthy controls. The results were complex. In the first experiment, patients with right PFC had more difficulty recognizing both basic and complex emotions compared to controls. In contrast, patients with left PFC damage had significantly more difficulty recognizing complex versus basic emotions compared to controls. When assessing for emotion type and valence, the right PFC patients did not reveal any statistically significant main effects or interactions, whereas the left PFC patients showed main effects for both emotion type and valence stimuli without a significant interaction. In the tachistoscopic experiment, the left visual field (right hemisphere) was significantly more accurate in recognizing basic emotions compared to the right visual field (left hemisphere). For complex emotions no difference was observed for visual field. When analyzing the tachistoscopic data based on valence, the right hemisphere compared to the left hemisphere had a marked advantage in recognizing basic negative emotions but there were no hemispheric differences in recognizing positive basic emotions. For the complex stimuli, there was no hemispheric differences in recognizing either positive or negative emotions. Thus, it was concluded that both experiments supported the ETH and VH but the VH was operative for basic rather than complex emotions.

The only other line of research that was formulated based on the ETH involves the neurological basis for the perception and expression of facial expressions. Although the research has not yet been applied to directly address the validity of the ETH, its importance relates to assumptions researchers have made when carrying out deductive experiments to support the RHH or VH and how the assumptions may have biased their results and conclusions.

Analysis of facial expressions has been a traditional means for inferring hemispheric lateralization of emotions by measuring expressive differences between the left and right hemiface based on the assumption that the right hemisphere controls the left side of the face and the left hemisphere controls the right side of the face [186,187,188,189,190,191]. The research, in general, has shown that the left hemiface is more expressive than the right hemiface in support of the RHH [192]. A meta-analysis of 16 research publications statistically confirmed that the left hemiface is more expressive than the right hemiface for posed but not for spontaneous facial expressions. However, the average effect size for the posed studies was relatively small and behaviorally weak, explaining only 3.6% of the data variance, indicating that, perhaps, facial expressions are not functionally organized across the vertical axis [186]. In fact, less well-known research has suggested that facial expressions are functionally organized across the horizontal facial axis with the lower face being better at expressing happy-pleasant and disgust types of emotions and the upper face being better at expressing surprise-fear, anger and sad types of emotions [193,194,195,196,197]. In addition, facial blends of emotion are a common phenomenon and have been observed in infants as early as 2 months [198,199,200,201,202]. Facial blends are an intrinsic element of display rules [169,170] whereby individuals may produce a “false” social smile to enable approach behaviors but may briefly leak an emotional expression on their upper face that better reflects their true feelings [169,170,171,172,173]. Facial blends may also occur if an individual experiences two competing emotions during a social situation [196,203].

In order to determine if there are hemispheric differences in the perception of upper versus lower facial expressions, Prodan et al. [204], using tachistoscopic techniques, presented drawings depicting three types of facial expressions to 30 healthy, right-handed, individuals: full facial expression, hemifacial expressions that just involve the upper or lower face and facial blends of emotion. The stimuli were randomly presented to either the right or left visual fields under two conditions: no instructions regarding where to direct visual attention and instructions to attend to the upper face. The subjects were asked to identify what emotion they perceived using a forced-choice response (happy, sad, angry, surprise, fear and neutral). Without attend instructions, the subjects overwhelmingly identified the lower facial expressions in either visual field when presented as a facial blend, suggesting that their intrinsic perceptual bias was to focus on the lower face. During the attend condition, the subjects robustly switched their perceptual bias to the upper face when the stimuli were presented to the left visual field (right hemisphere). When the stimuli were presented to their right visual field (left hemisphere), their perceptual bias to the lower face lessened but did not fully shift to the upper face. Thus, it was concluded that the right hemisphere was associated primarily with perception of upper facial emotions. The perceptual bias to lower facial expressions under the no attend condition was attributed to three possibilities: (1) subjects normally attend to the lower face to enhance verbal comprehension, especially in noisy environments (“McGurk effect”) [205,206,207], (2) due to cultural norms, subjects focus their visual attention to the lower face in order to avoid direct eye contact which is perceived as being aggressive and threatening in humans (“evil eye”) and other animals, unless there is mutual affiliation [208,209,210,211], and (3) the left-hemisphere is more involved with foreground (conscious) processing of visual information whereas the right hemisphere is more involved with background (subconscious) processing of visual information [212,213] that can be brought to the foreground by altering an individual’s attentional bias. This conscious-subconscious dichotomy also appears to extend to the perception and processing of emotional information conveyed by facial expressions and affective prosody during social interactions [214,215] but not for intense emotional experiences that may bring the right hemisphere’s perceptions to the foreground [1].

In a follow-up publication [216], 20 strongly right-handed individuals were assessed for their ability to pose full facial expressions versus upper-lower and right-left facial blends of emotion. The impetus for the study was the pivotal research by Morecraft and colleagues [217,218] showing that in monkey, and presumably humans, the lower face was neuroanatomically controlled by the primary and premotor cortices residing in the posterior, infero-lateral, frontal lobe whereas the upper face was neuroanatomically controlled by motor areas residing in the posterior-medial frontal lobe (supplementary motor area and anterior cingulate gyrus). The subjects rated their degree of posing difficult for the various tasks using a 5-point Likert scale and judges rated how well the subjects actually posed the requested facial expressions. The results were statistically very robust, indicating a strong behavioral effect. As expected, full facial emotions were the easiest and most accurate to pose. Right-left facial blends were the most difficult and most unnatural to pose and were judged as least accurate. In contrast, upper-lower facial blends compared to right-left facial blends were relatively easy to pose and judged as more accurate, lending support to the concept that facial expressions are functionally organized across the horizontal rather than the vertical axis of the face [186,202].

To further elucidate the motor physiology underlying facial expressions, Ross and colleagues [202,219,220], using high-speed videography (600 frames per second), assessed the movement dynamics of posed and spontaneous facial expressions. The initial goal of the research was to determine if movement onset asymmetry was a more powerful method to determine the hemispheric origin of a facial expression compared to the traditional use of expression intensity as measured by the magnitude of terminal movement asymmetry. The initial, “proof of concept”, publication [219] assessed the movement dynamics of three facial expressions (smile, frown, surprise) in 20 healthy, right-handed, individuals under posed and spontaneous conditions. Fiducial markers (black bindis) were placed on the subject’s face at the corners of the mouth, above the mid eyebrows, on the upper cheeks adjacent to the nose and mid chin. The bindis served as land marks for following facial movements over time using a video editing program (Premiere Elements 7, Adobe Systems, Inc.) in which frame to frame movement of the bindis could be assessed by hand, a procedure that was both tedious and time consuming. In the subsequent publications [202,220], the bindi movements were analyzed using a computer-based program that was considerably more efficient. The results were statistically quite robust, explaining up to 70% of the data variance. Of the 111 facial expressions analyzed, 96 showed movement-onset asymmetries. Posed expressions began principally on the right side of the face (42 of 48, 87.5%), indicating a left hemisphere origin, whereas spontaneous expressions began principally on the left side of the face (43 of 48, 89.5%), indicating a right hemisphere origin. The results were strongest for the upper facial expressions and less so for smiling. Surprisingly, movement onset asymmetry was not statistically predictive of terminal movement asymmetry and terminal movement asymmetry did not show a statistically significant lateralized effect. Thus, it was concluded that movement-onset asymmetry was a more powerful method for inferring the hemispheric origin of a facial expression compared to terminal movement asymmetry (expression intensity).

The next publication [203] (part I of II) explored, in detail, the neurophysiological basis of spontaneously induced facial blends of emotions. Forty-five, right-handed, individuals participated in the study. They viewed four short video clips designed to elicit either a social and/or primary emotional reaction, similar to the approach used by Buck and Duffy [1] in their publication establishing that display rules are modulated primarily by the left hemisphere [176]: (1) a sitting infant who laughs so hard that he falls over (social > primary), (2) models walking across a stage with one unexpectedly falling into a small square opening in the stage (primary > social), (3) a traffic video of a bus careening out of control that narrowly misses a pedestrian(primary > social) and (4) a precision marching team that crisscrosses paths at a walk, double time and walking backwards at double time (social > primary). There were 307 examples of temporally distinct upper and lower facial expressions: 102 smiles, 25 grimaces, 46 frowns, 7 snarls and 47 spontaneous expressions that were categorized as “vocalics.” e.g., “mmmmmh”, “woooow”, uuuuuh”. Of the 307 expressions, 144 were identified as being associated at some point in time with a contrahorizontal facial expression that ultimately yield 82 examples of unique facial blends. Each expression comprising the facial blend was analyzed for onset movement asymmetry, timing and magnitude to the movement peak and timing to the end of the expression. Using these measures, the upper and lower facial expression was classified as being motorically independent of each other based on meeting one or more of the following criteria: (1) the initiating expression started on one side of the face and the contrahorizontal expression started on the other side of the face, (2) the initiating expression started and peaked before the contrahorizontal expression started and peak, (3) the initiating expression started after the contrahorizontal expression and peaked before the contrahorizontal expression peaked, (4) the initiating expression started and peaked before the contrahorizontal expression started and peaked, (5) the initiating expression started, peaked and ended before the contrahorizontal expression peaked, or (6) the second expression started after the contrahorizontal initiating expression but its movement velocity was greater and it peaked before the contrahorizontal expression. Using the above criteria, 79 (95%) facial blends were found to have independent motor control and 3 (4%) were found to possibly have dependent motor control: the contrahorizontal expression started after and lagged behind the movement of the initiating expression causing it to peak and end before the initiating expression. Statistically, this outcome was extraordinarily robust, explaining 86% of the data variance. In addition, 12 facial blends had either an upper or lower expression that was strictly unilateral, two facial blends were composed of a strictly unilateral right-sided expression in combination with a strictly left-sided contrahorizontal expression and four “double” blends were encountered in which the contrahorizontal expression was composed of two different expressions over time. Of the 12 facial blends in which one expression was strictly unilateral, four contrahorizontal expressions began on the opposite side of the face. Thus, it was concluded that the motor control of upper and lower facial expressions in humans was motorically independent, consistent with the neuroanatomical findings in monkeys published by Morecraft et al. [218,219]. In addition, spontaneous facial expressions appear to be motorically complex rather than monolithic entities, supporting the Component Theory of facial expressions [221,222,223] rather than the Primary Emotional theory of facial expressions [6,7,9,224,225].

The second publication (part II) [220] explored the neurophysiological basis of spontaneously induced expressions involving either the upper and lower face that excluded the “vocalics” mentioned above (99 smiles, 25 grimaces, 70 surprises, 45 frowns, 7 snarls, 5 facial blends that occurred across the *vertical* facial axis). In total, 251 facial expressions were analyzed by determining movement-onset asymmetry, graphing and quantitating the vector movement of each side of the face to its peak and measuring movement velocity. Four unique phenomena were observed. There were five instances of a facial blend occurring across the *vertical* facial axis (4 grimace-smiles, 1 surprise-frown). Thirty-two expressions (16 smiles, 2 grimaces, 7 surprises, 7 frowns) were initiated on one side of the face but the movement was motorically taken over by the other side of the face (takeover phenomenon), indicating that the seemingly “unitary” expression was actual the result of motorically independent or competing innervations emanating from *both* hemispheres that produced a non-traditional facial blend of emotion in which the blend consisted of the *same* expression. There were 36 expressions that showed two or more discrete legs to the movement that were classified as a “seesaw” phenomenon. Each leg was analyzed separately. If the legs showed a different start-side and/or a takeover phenomenon, then the seemingly “unitary” facial expression was classified as being motorically independent. Of the 36 seesaw expressions, 34 met the criteria of independent or dual hemispheric innervation (22 smiles, 2 grimaces, 4 surprises, 6 frowns) producing a non-traditional facial blend of emotion. There were 17 expressions that were strictly unilateral (4 smiles, 4 grimaces, 6 surprises, and 3 frowns) that were also classified as showing independent hemispheric motor control. If the unitary expression was deemed to be motorically dependent (no evidence for dual hemispheric innervation), 151 of 154 (98%) expressions also exhibited maximal facial movement on the same side of the face. If the unitary expression was deemed to be independent (dual hemispheric innervation), 40 of 62 (65%) expressions exhibited maximal facial moment on the contralateral side of the face, a finding that possibly explains why start side is not necessarily predictive of side of maximal facial movement as reported in the “proof of concept” publication [219]. Based on the results found in both papers, it was concluded that spontaneous facial expressions are functionally organized primarily across the horizontal facial axis and secondarily across the vertical facial axis and that spontaneous facial expressions are complex, multi-component rather than monolithic, motoric events; thus, lending strong support for the Component Theory of facial expressions [221,222,223] and emotions [226] that “…best explains the neurobehavioral imperatives underlying facial expressions in adults who have fully developed language and communication skills, representational memory and cognitive schemas, including display rules, emotional appraisal and attribution” (page 40) [202]. What this mean is that when adults react to an environmental event they may simultaneously experience and facially express more than one emotion at a time, resulting in facial blends across the vertical and/or horizontal facial axis.

In terms of emotional lateralization, the results of the two studies do not support either the RH or VH. Although the studies were not designed to determine the validity of the ETH, the results indirectly support the ETH. Facial blends that display different emotions across the horizontal or occasionally across the vertical facial axis could be interpreted as representing a primary emotional response, if the expression started on the left side of the face (right hemisphere), and the contrahorizontal or contralateral expression could be interpreted as representing a social emotional response, if the expression is initiated on the right side of the face (left hemisphere). For the seemingly “unitary” expressions that occur on the upper or lower face that show dual hemispheric innervation, one could interpret that the right-sided expression (left hemisphere) represents a social emotional response whereas the left-sided expression (right hemisphere) represents a primary emotional response, even though both sides of the face are functionally displaying the “same” emotional expression that may be classified as either negative or positive in valence. For example, an individual can be surprised by meeting an old friend by chance at an airport and simultaneously be surprised that the friend appears to be chronically ill or a parent may frown at a child for socially misbehaving and simultaneously frown because the child punched his sibling. If a unitary expression does not demonstrate dual hemispheric innervation, then it could be interpreted as a social emotional response, for example, a “false” smile to enable social interaction versus a truly genuine social smile on meeting a valued colleague or, perhaps, a grimace when a student receives his failing grade on a mid-term examination, if it is initiated on the right side of the face (left hemisphere) regardless of its valence. If the unitary expression is initiated on the left side of the face (right hemisphere), for example a frown or a smile, then it could be interpreted as a primary emotional response regardless of its valence. Finally, anger is typically categorized as a primary emotion. However, if an angry expression is initiated on the right side of the face, it could be interpreted as a social rather than a primary emotional response to a life event, e.g., an individual can be angry because her male supervisor would not support her corporate promotion.

## 7. Conclusions

The neurology underlying emotions and related display behaviors outlined in this paper powerfully support the concept that emotions are experienced, processed and cognitively appraised by *both* hemispheres, thus invalidating the RHH of emotional lateralization. The neurology also suggests that VH does not fully explain the differential hemispheric lateralization of emotions and related display behaviors. Emerging data suggests that the ETH may be a more effective means to approach the issue of emotional lateralization. However, only future deductive types of research will be able to definitively validate the ETH provided that appropriate stimuli and response measures are utilized. For example, research has established that patients with right hemisphere damage have markedly diminished autonomic responses to emotional stimuli compared to patients with left hemisphere damage, a finding that has been used to support the RHH [138,139,140,141]. However, if the stimuli used to induce the autonomic responses were primary emotional in quality that did not specifically include social emotional stimuli, then the results would be substantially biased to find right but not left hemisphere autonomic hyporeactivity. Similarly, the serendipitously observed changes in emotional memories during the right-sided Wada test that was the basis for formulating the ETH [1] were based on recall of a primary emotional life event. If the research were to be done as a deductive inquiry to test the validity of the ETH, two life events should be identified, one associated with a strong primary-emotional memory and one associated with a strong social-emotional memory, before the patient undergoes a right-sided Wada test. There has also been some interesting research looking at emotional lateralization in patients who underwent partial or full surgical resection of their corpus callosums for control of intractable epilepsy [227,228]. However, the impetus for the research was based on validating the VH and/or RHH. Assuming that patients with callosotomies have fairly normal intellectual development, they could serve as excellent neurologic subjects to validate the ETH, if they are assessed with appropriate emotional stimuli.

The last point regards the use of functional imaging for localization purposes. Over the past 40 years, functional imaging has replaced the traditional lesion-based method for defining functional-anatomic relationships [229,230]. However, due to serious methodological problems, functional imaging has produced a myriad of localizations that are often not confirmed by traditional lesion-based research and does not show areas in the brain that are known to be critically involved in a cognitive-behavioral function that is instantiated as a distributed neural network [19,20,215], which has led to the conclusion that most functional imaging is essentially neurophysiologic “phrenology” [231,232,233]. Therefore, any research using functional imaging as a means to determine the neuroanatomical basis of a cognitive-behavioral function, including emotions [234], needs to be viewed very skeptically unless the localizations have been confirmed using traditional lesion-based research methods, which also has its own limitations that must be taken into account [19,20,215].

## Data Availability

Not applicable.
